# Complementary Medicine and Self-Care Strategies in Women with (Recurrent) Urinary Tract and Vaginal Infections: A Cross-Sectional Study on Use and Perceived Effectiveness in The Netherlands

**DOI:** 10.3390/antibiotics10030250

**Published:** 2021-03-03

**Authors:** Louise Witteman, Herman A. van Wietmarschen, Esther T. van der Werf

**Affiliations:** Louis Bolk Institute, Kosterijland 3-5, 3981 AJ Bunnik, The Netherlands; L.witteman@louisbolk.nl (L.W.); h.vanwietmarschen@louisbolk.nl (H.A.v.W.)

**Keywords:** urinary tract infection, vaginal infection, antibiotics, antifungals, AMR, patient’s perceived effectiveness, complementary medicine, self-care

## Abstract

Due to the excessive use of antibiotic and antimycotic treatments, the risk of resistant microbes and fungi is rapidly emerging. Previous studies have demonstrated that many women with (recurrent) urinary tract infection (UTI) and/or vaginal infections (VIs) welcome alternative management approaches to reduce the use of antibiotics and antifungals and avoid short- and long-term adverse effects. This study aims to determine which complementary medicine (CM) and self-care strategies are being used by women suffering from (recurrent) UTI and VI in The Netherlands and how they perceive their effectiveness in order to define directions for future research on safety, cost-effectiveness, and implementation of best practices. A cross-sectional online survey was performed among women, ≥18 years old, with a history of UTIs; 162 respondents were included in the data analysis, with most participants aged between 50 and 64 years (36.4%). The women reported having consulted a CM practitioner for UTI-specific symptoms (23.5%) and VI-specific symptoms (13.6%). Consultations of homeopaths, acupuncturists, and herbal physicians are most often reported. Overall, 81.7% of the women suffering from UTI used complementary or self-care strategies besides regular treatment, and 68.7% reported using CM/self-care strategies to treat vaginal symptoms. UTI- related use of cranberries (51.9%), vitamin C (43.8%), and D-mannose (32.7%) were most reported. Perceived effectiveness was mostly reported for homeopathic remedies and D-mannose. The results showed a substantial burden of UTI and VI on daily and sexual activities. Besides the frequency of use, the indication of perceived effectiveness seems to be an important parameter for further and rigorously designed research to encourage nonantibiotic/antifungal treatment implementation into daily clinical practice.

## 1. Introduction

The discovery of antibiotics is one of the most important events in medical history, and it would have added a decade to people’s life expectancy [1]. However, as a result of many years of inappropriate use (under- and overuse) of antibiotics, there is currently a huge problem of antibiotic-resistant microbes, causing infections that are very difficult to treat [2]. Antimicrobial resistance (AMR) and inappropriate use of antibiotics pose a serious national and international threat to public health. Reducing the use of antibiotics to control the development of AMR is, therefore, an urgent national and international priority [3]. Around 75% of all health service antibiotics are prescribed in primary care [4]. In primary care, there are concerns that some common infections are becoming increasingly difficult to treat and that illnesses due to antibiotic-resistant bacteria may take longer to resolve. In The Netherlands, urinary tract infections (UTIs) are the most common reason for women to consult a GP, with 232 out of 1000 contacts being UTI-related [5]; UTIs are an important reason for a GP to prescribe an antibiotic [6]. Routine primary-care prescribing of antibiotics is associated with an increased risk of antibiotic-resistant UTIs in adults [6] and children [7]. Half of all women experience at least one urinary tract infection (UTI) during their lifetime [8]; up to 30% of them experience at least one recurrence, and around 25% of these develop ongoing recurrent episodes [8]. Recurrent UTIs (rUTIs) have negative implications for quality of life [8,9] and healthcare costs, including costs of primary care consultations, antibiotic prescribing, outpatient visits, and diagnostic tests [4]. In addition to the problem of AMR, using antibiotics is found to have short- and long-term side effects for the individual too, such as diarrhea, rashes, candidiasis, and microbiome disruption [10], and may be associated with depression, anxiety, psychosis [11], immune dysfunction [12,13], Parkinson’s disease, obesity, and diabetes [14]. Although antibiotic therapy for UTIs may be seen by many GPs as the most effective treatment, as few as 25% of women with urinary symptoms may have a culture-proven bacterial infection, and many with symptoms of uncomplicated UTI recover without antibiotic treatment [15,16]. Previous studies have demonstrated that many women with rUTIs would welcome alternative management approaches to reduce the use of antibiotics and avoid short- and long-term adverse effects [10,14]. Therefore, it is important to stimulate the use of alternatives to antibiotics that would reduce UTI incidence, costs, and antibiotic use, while improving antimicrobial stewardship.

In addition to AMR, fungi can also develop resistance to antifungals [17]. Fungal pathogens can cause life-threatening invasive diseases (e.g., fungemia, meningitis, pneumonia), severe chronic conditions (e.g., chronic pulmonary aspergillosis, allergic bronchopulmonary aspergillosis), and complex chronic respiratory conditions (e.g., asthma, chronic obstructive pulmonary disease) [18]. These pathogens also cause (recurrent) infections, such as oral and vaginal candidiasis [18]. Candida infections are most often caused by *C. albicans*, as shown by epidemiological studies in the United States [19], Europe [20], and the Middle East [21]. Vulvovaginitis, or vaginitis, is frequently cited as the most common reason that women visit their primary care providers [22], and vaginal symptoms have a negative impact on patients in terms of discomfort and pain, days lost from school and/or work, sexual functioning, and self-image [23,24]. The most common causes of infectious vaginitis are bacterial vaginosis (BV), vulvovaginal candidiasis (VVC), and trichomonal vaginitis (TV) [25]. Past research has shown women often feel dissatisfied with the clinical care they receive when presenting with vaginal symptoms [26,27], and both patients and clinicians frequently misdiagnose the symptoms [26]. The most commonly prescribed antifungal used for *C. albicans* infections is fluconazole, to which resistance is rising [27].

The emergence of antibiotic and antifungal resistance asks for an urgent broader view on possible solutions. The use of complementary therapies (CM) could be one direction for novel solutions. A web-based analysis performed by Flower et al. [28] revealed that some women express dissatisfaction with the side effects and short-term benefits of antibiotics. As a consequence, complementary therapies are already widely used by women on the forum as primary or adjuvant treatments to alleviate symptoms and to address the perceived deeper immunological or emotional causes of these infections [28]. Additionally, the contribution of honey in alleviating vaginal discomfort caused by Candida infections has already been demonstrated in various in vivo studies [29,30,31]. However, there is a need for more rigorous assessments of alternatives to conventional treatments, such as these CM interventions, as well as for information on the perceived effectiveness of these interventions.

The aim of this study is to determine the use and patient-perceived effectiveness of CM and self-care strategies for (recurrent) urinary tract infections and vaginal infections in women in The Netherlands in order to define directions for future research on safety, cost-effectiveness, and implementation of best practices.

## 2. Method

A cross-sectional online survey on the use and patient-perceived effectiveness of CM and self-care strategies in women with (recurrent) urinary tract infections and vaginal infections was carried out in The Netherlands between June 2020 and October 2020. For this study, CM has been defined as a group of diverse medical and health care professionals, practices, and products that are not generally considered part of conventional medicine [32], and self-care includes nutrition and dietary supplements.

Female participants, ≥18 years old, with a history of UTI, were recruited on the internet. Study information and a link to the online survey on the Survey Monkey platform were shared on the Facebook pages: “interstitiele Cystitis Patienten Vereniging ICP” from the Interstitial Cystitis Patient Organisation, “Eeuwige blaasontsteking”, “Vrouwenpoli”, and on the Instagram account “Vrouwenpoli” from the Ellesie Female Clinic, The Netherlands.

The survey consisted of 36 single-choice and multiple-choice questions (including an open question for any additional comments), including questions about demographics (3 questions), incidences of UTI/VI (4 questions), consultation and treatment of UTI/VI (16 questions), and perceived effectiveness of self-care/CM treatment options (6 questions), as well as questions regarding the impact of suffering from UTI/ VI on daily activities, sexual activities and mood (6 questions). The survey had been piloted to test the validity and reliability of the tool. The demographic characteristics collected were age, living area, and level of education. Education was classified as higher education ((applied) university/postdoctoral level), secondary education (middle and higher secondary education), and lower education (no school/primary school only/lower secondary education). Recurrent UTIs were defined as a minimum of 2 (self-reported or physician-confirmed) UTIs in 6 months or a minimum of 3 (self-reported or physician-confirmed) UTIs in 12 months [33].

At the start of the survey, participants were informed of the anonymous and summarized reporting of their data. Completion of the survey was regarded as an indication of participant’s consent to the study. All data were anonymously collected and reported. The anonymous nature of the web-survey did not allow us to trace, in any way, sensitive personal data. The study protocol was reviewed by the Medical Ethical Reviewing Committee of Wageningen University, The Netherlands. A letter was received from the committee (dated 17 June 2020), stating that the study did not fall within the remit of the Dutch Medical Research Involving Human Subjects Act (WMO) and was, therefore, exempt from further medical ethical review.

## 3. Statistical Analyses

Descriptive statistics such as measures of central tendencies, frequencies, and proportions were used to evaluate the responses. Data are represented as numbers and/or percentages for categorical variables or mean and standard deviation for continuous variables. Pearson’s chi-square tests were performed to identify differences in sociodemographics (age and education level) and CM use in general and for UTI/VI specifically. For these analyses, age was recoded into two age groups (18–49 and ≥50), education was recoded into “lower to secondary” education and “higher” education. Statistics were carried out using Statistical Package for Social Sciences (SPSS) v. 26.0. Results were considered statistically significant with a *p*-value < 0.05.

## 4. Results

A total of 204 women completed the online questionnaire. After validation of the data, 162 respondents were included in the study. Respondents who were younger than 18 years old (*n* = 1) or did not complete the questions on baseline characteristics (*n* = 7) were excluded. In addition, respondents without a history of at least one physician-diagnosed UTI were excluded from the dataset (*n* = 34). Another 21 respondents filled out their date of birth incorrectly, resulting in their age being classified as unknown. Of the 162 women with a history of at least one physician-diagnosed UTI, 80 women had a history of VI as well.

As shown in Table 1, most respondents were between 50 to 64 years old (36.4%), followed by 27.2% between 30 and 49 years old. Respondents were distributed across the 12 provinces, with 29.6% from the northern regions of The Netherlands, 43.2% from the central regions of The Netherlands, and 27.2% from the southern regions of The Netherlands. Almost all (98.8%) of them had finished at least primary education. One-third (30.8%) of the respondents indicated the use of CM in daily life (not UTI/VI-specific). Consulting a homeopath (11.7%) was most frequently reported, followed by consulting an acupuncturist (10.5%) and/or an herbal physician (8.6%).

### 4.1. Incidence, Consultation, and Treatment of UTI and VI

Of the 162 women, 48.8% (*n* = 66) suffered from rUTIs (a minimum of 2 (self-reported or physician -confirmed) UTIs in 6 months or a minimum of 3 (self-reported or physician-confirmed) UTIs in 12 months); 41.8% of all respondents received two or more treatments for UTI symptoms over the past two years, while 35.1% did not receive any treatment. More than half of the women with a history of UTIs (56.4%) also received treatment for VIs in the past 2 years; 81.7% of our study population used CM or self-care to treat UTI symptoms, while 68.6% used CM or self-care to treat their vaginal symptoms (Table 2).

When visiting their GP, about half of the women indicated that they expected to receive antibiotics or antimycotics to treat their UTI or VI (50.0% and 46.25%, respectively). Table 2 shows homeopaths, acupuncturists, and herbal physicians to be the most consulted CM practitioners for UTI- or VI-related symptoms (respectively, 17.9%, 8.6%, and 6.7% for UTI and 15%, 8.8%, and 7.5% for VI).

Women who seek advice on CM/self-care strategies for UTI/VI most often use the internet (42.0%) to gather information. In addition to the internet, women ask their GP/treating consultant (21.0%) or friends and family (18.5%) for advice. Only 6.2% of the women rely on patient associations for advice on UTI-specific CM/self-care strategies, and only 5% rely on patient associations for advice on VI-specific CM/self-care strategies. For both UTI and VI, women additionally reported seeking advice on orthomolecular medicine (4.3% and 3.75%, respectively).

### 4.2. Use and Perceived Effectiveness of CM and Self-Care Strategies

Age, education, and history of recurrent UTIs were not statistically significantly associated with CM/self-care use in general (χ2 = 0.8, *p* = 0.36; χ2 = 0.020, *p* = 0.90; χ2 = 3.2, *p* = 0.36, respectively), UTI-specific CM/self-care use (χ2 = 0.1, *p* = 0.71; χ2 = 0.03, *p* = 0.87; χ2 = 1.6, *p* = 0.67, respectively), and VI-specific CM/self-care use (χ2 = 1.4, *p* = 0.24; χ2 = 0.04, *p* = 0.85; χ2 = 0.1, *p* = 0.79, respectively).

Table 3 reports on the use of CM and self-care strategies instead of or in addition to women’s regular treatment for UTIs and VIs. About 50% of the women suffering from UTIs use cranberry juice or cranberries as a self-care strategy to treat the infection (with or without visiting a doctor). Other popular products are vitamin C (43.8%) and D-mannose (32.7%). Probiotics, homeopathic remedies, and general painkillers are also often reported to be used. The majority of the women indicated using at least three different products. The combination of cranberry/D-mannose and probiotica was used by 20 respondents. Chinese/Eastern herbal medicine was not very commonly used (*n* = 5). About half of the women (51.2%) who used homeopathic remedies for UTIs reported this treatment as effective (versus 12.2% reporting the remedy not to be effective). Positively perceived effectiveness for UTI symptom relief was also frequently reported (43.3%) for the use of D-mannose.

Women suffering from VI most often use antimycotic cream (*n* = 31) and report this as being effective in 45.2% of the cases; 29 women reported the use of probiotics (which was perceived to be effective by 17.2% of the cases), and 15 women indicated the use of a vaginal shower (although none of them perceived this as effective). Only 11 women used homeopathic remedies; however, over 50% of these women indicated the homeopathic remedy as effective, and none of them perceived it as not effective. Very few women (*n* = 4) used acupuncture as CM treatment for their VI, but 3 out 4 respondents perceived this treatment as effective.

### 4.3. Interference with Daily Life

Table 4 shows that suffering from UTI may have serious consequences for daily activities: the women reported to be often restrained in their sexual activities (41.9%), going outside (29.0%), and sports activities (24.5%). It also affects basic needs such as sleep, social activities, and work. Women suffering from VI also often experience inconvenience during sexual activities (34.5%), cycling (24.6%), and sleeping (18.3%). Moreover, social activities and sports can be hindered, and it negatively impacts the motivation to undertake activities. Of the women indicating that sexual activities are affected by UTIs (*n* = 82), 28.0% reported not to be in the mood for sex anymore, 25.9% experience hassle and pain, and 22.5% had no sex anymore. Some respondents further explained in the open answer category that they have less or no sex to protect themselves from recurrent UTIs, which might indicate that sexuality is affected in general and not only during an actual infection. Within the VI-group, 21.4% (12 out of 56) did not have sex anymore, and 21.2% did not have sex spontaneously or experienced pain (11 out of 52), closely followed by not being in the mood for sexual activities (19.2%; 10 out of 52).

## 5. Discussion

This study aimed to determine which complementary medicine and self-care strategies are used by women suffering from (recurrent) UTIs and VIs in The Netherlands and how the effectiveness of those strategies are perceived. More than three-quarters (81.7%) of the women suffering from UTIs and more than two-thirds (68.7%) of women suffering from VIs indicated using CM/self-care strategies. Cranberries, vitamin C, and D-mannose are most commonly used to self-treat UTIs, with a high percentage of women indicating a strong perceived effect when using D-mannose. VIs are mostly self-treated by using antimycotic creams, probiotics, and/or vaginal showers, although, for the latter, the perceived effectiveness is lacking. The extent to which UTIs and VIs are reported to affect women’s daily life activities, especially sexual activity, is striking. This study also indicates that about half of the women consulting their GP are still expecting to receive antibiotics to treat their UTI or antimycotics for VI. Besides frequency of use, the indication of perceived effectiveness of the reported CM/self-care strategies seems to be an important parameter for further and rigorously designed research to encourage nonantibiotic/antifungal treatment implementation into daily clinical practice.

As stated in the Dutch guidelines on the treatment of UTI [35], antibiotics is the first choice of treatment for women with UTIs [35,36]. Although CM and self-care strategies seem widely used in addition to conventional treatment by women with rUTIs, in line with these guidelines, our study shows that half of the women consulting their GP for the suspicion of UTI expect to receive antibiotics to treat the infection. Our study shows that the information on CM and/or self-care strategies is mostly gathered from the internet, and that just one-fifth of the women reported being informed on these strategies by their healthcare professional. Previously, a lack of CM knowledge was found in The Netherlands [37], and not all Dutch GPs are known to be enthusiastic about CM treatments yet. This might be explained by the lack of knowledge about CM remedies, as was previously concluded based on a survey among Australian GPs. Almost all of the Australian GP respondents stressed the need for more scientific testing before remedies could be implemented in conventional healthcare [38].

We found cranberry juice or cranberries to be the most commonly reported self-care method for UTI treatment. About a quarter of the women perceived cranberry juice/cranberry-containing products to be effective, whereas another quarter did not notice any effect. This might be explained by the fact that there is a substantial group in this study who uses the remedy as curative instead of preventive. Since the data in this study is too limited to clearly distinguish between curative and preventive use, it might be worth investigating whether cranberry juice and cranberries can best be used as preventive, curative, or both. However, the effectiveness of cranberries for the prevention or treatment of UTIs is still under scientific debate. Luis et al. concluded, based on a meta-analysis and sequential analysis of clinical trials, that the use of cranberry has a clear positive effect on the prevention of recurrent UTIs [39]. Thus far, few studies have assessed the potential benefit of cranberry in treating symptoms of rUTIs or whether cranberry might have a synergistic effect when combined with antibiotics [2,40,41]. If cranberry safely and effectively treats UTIs or acts synergistically with antibiotics, this could substantially help to reduce overall antibiotic exposure. Future well-designed studies, in line with, for example, the CUTI feasibility study [42], are warranted.

The second most-used self-care method in our study population is vitamin C. The number of women who find this remedy effective is similar to cranberry; however, no clinical evidence is available to support the use of vitamin C to prevent or treat rUTIs [43].

Although the number of studies is small, a recent systematic review and meta-analysis concluded that D-mannose protects against rUTIs and its effectiveness seems similar to antibiotic treatment [44]. In our study, D-mannose was found to be the third most commonly reported self-care strategy. Over 40% of its users reported D-mannose as effective, which supports the findings of the meta-analysis [44].

Probiotics have also been reported by our participants with regards to UTIs, but the reported perceived effectiveness, based on the small number of users, was not convincing. This is in line with the literature stating that so far, no strong evidence in favor or opposed to the use of probiotics has previously been found [45]. The often-reported combination of products, “probiotics, cranberries, and D-mannose”, might reveal the need for evidence on the effectiveness and safety of already existing combination products.

The current study shows the homeopath to be the most popular CM health practitioner to be consulted in general, as well as for UTI and VI specifically. However, only 25% of the women used homeopathic remedies to treat UTI, with only 14% using them to treat VI. Interestingly, the majority of homeopathic remedy users perceived the treatment as effective (over 50% for both indications). The number of convincing good-quality studies supporting the evidence of homeopathy is scarce. More pragmatic research should be performed to determine the effectiveness of homeopathic treatment for UTI /VI compared to or in addition to regular treatment.

Some treatment options were only very little reported in our study, but, in contrast to frequency, the reported perceived effectiveness was rather high. For example, acupuncture for treating VI was rated as effective by 3 out of 4 respondents as well as neutral to highly effective for treating UTI (neutral by 6 out of 11, and effective by 3 out of 11). This latter finding is in line with a recent review hinting towards the positive effects of acupuncture on recurrent UTI [46]. The same holds for Chinese/Eastern herbal medicine, the use of which was only very little reported in our study but was perceived to be effective in treating UTI by 2 out of 5 women. This potential benefit has previously been described in the meta-analysis of Flower et al. [28]. Future high-quality pragmatic clinical trials to determine the (cost) effectiveness of these CM treatments as alternatives to antibiotic treatment or to delay antibiotic prescription rates are warranted.

Less common but interesting self-care methods that were mentioned by some respondents were drinking baking soda, orthomolecular therapies, mesology, pelvic floor therapy, and the use of a limited-sugar diet. Future systematic literature reviews should include these modalities to provide more knowledge on the existing evidence.

When considering popular self-care options to treat VI, it is interesting that probiotics are quite often used, although only 17.2% of its users reported the treatment as effective. A Cochrane review concluded that evidence of its effectiveness in VI treatment is not yet convincing; hence, more well-designed studies are needed [47]. Recently, it was also concluded that data about the effectiveness of probiotics in treating VI is not very consistent, although there is a lot of advertising for this remedy [48], which might explain why its use is popular.

Another strategy that is frequently used to treat VI is a vaginal shower. None of our respondents reported this as effective, which is consistent with previous studies concluding that vaginal showers with lactic-acid-containing products [49] or with just water might even increase the chance of developing a VI [49,50] and that showering might do more harm than good [51,52]. Again, it is possible that marketing strategies explain the popularity of these products.

Several strengths and limitations of this study need to be noted. Our study has been strengthened by the fact that the survey focused not only on the use of CM and self-care strategies but aimed to explore the perceived effectiveness of these strategies as well. This information provides the foundation for future research, where safety (synergetic use), (cost)-effectiveness, implementation, and evaluation should be central. Real-world evidence (RWE) data might be a very helpful source for discovering promising strategies. Most survey answer categories included an open-text category that motivated the respondents to respond and thereby strengthened our study by exploring all additional CM and self-care strategies being used.

Our results are limited by the use of a self-reported questionnaire. The assessment of CM and self-care strategies was based on individual recall methods and not by direct measurement of use. Respondents may, thus, have either overestimated or underestimated the perceived effects. The reported perceived effectiveness of the different strategies used is subjective. It cannot be concluded whether (r)UTIs were successfully treated by the chosen complementary remedy or if the (r)UTI could be healed on its own or by having conventional treatment only. Additionally, some respondents used multiple remedies at once, so the effectiveness could not be attributed to one specific remedy. This study does not aim to demonstrate the differences in effectiveness among several strategies or between preventive and curative strategies; nevertheless, this study supports the idea that there are alternatives to antibiotics and antifungals that are perceived to be effective. An obvious other limitation of a cross-sectional study design is that it does not allow causal inferences about relationships and, thus, limits any claim about the directionality of the results.

Since the recruitment of subjects was targeted to women with a history of UTIs and took place via selected social media, we cannot exclude the possibility of overrepresentation of women with severely complicated UTI symptoms, which might influence the representativeness of our study population. CM and self-care use and perceived effectiveness might therefore differ in women with only acute, noncomplicated UTIs. This survey was performed on the general public, not on patients; therefore, differentiating between women with UTI symptoms or culture-proven UTI in relation to CM use cannot be determined in this study. For future studies, determining the use of CM and their perceived effectiveness in women consulting their GP with UTI symptoms would be of interest to differentiate between women with UTI symptoms and women with a culture-proven UTI.

Fortunately, in the past years, there has been growing interest in the use of nonantibiotic treatments for common infections, such as UTIs, in order to reduce AMR. Our findings emphasize the enormous impact of UTI and VI on daily and sexual activities and, thereby, support the importance and urgent need to further investigate nonantibiotic/antifungal treatment options. The reported effectiveness based on our study is subjective. However, the reported positive experiences highlight the need for more rigorously designed research on some promisingly reported CM and self-care strategies used in addition or as alternatives to antibiotics in order to encourage the implementation of these remedies in daily clinical practice. Future research should also focus on the impact of CM and self-care treatments on antibiotic prescription and use and take into account the perception of healthcare professionals on synergetic use.

## Figures and Tables

**Table 1:** Baseline characteristic and use of complementary medicine (CM).

	Total Study Population *n* = 162
	*n* (%)
**Age categories**	
18 to 30	8 (4.9)
30 to 50	44 (27.2)
50 to 65	59 (36.4)
65+	29 (17.9)
Not known *	22 (13.6)
**Region (The Netherlands)**	
Northern regions	48 (29.6)
Central regions	70 (43.2)
Southern regions	44 (27.2)
Education **	
Lower education	2 (1.2)
Secondary education	75 (46.3)
Higher education	85 (52.5)
**History of vaginal infections (VI)**	80 (49.4)
**Use of CM**	50 (30.9)
**Consultation CM practitioner (multiple answers allowed)**	
(Classical) Homeopath	19 (11.7)
Acupuncturist	17 (10.5)
Herbal physician (Western/Eastern)	14 (8.6)
General practitioner (GP) with anthroposophic vision ***	6 (3.7)
Traditional Chinese physician	6 (3.7)
Anthroposophical therapist	5 (3.1)
General practitioner (GP) with homeopathic vision	3 (1.9)
Other	14 (8.6)

* Filled out their date of birth incorrectly, resulting in age classified as unknown. ** Higher education ((applied) university/postdoctoral level), secondary education (middle and higher secondary education), and lower education (no school/primary school only/lower secondary education). *** Anthroposophic medicine is an extension of conventional medicine and incorporates a holistic approach to people and nature and to illness [33].

**Table 2:** Incidence, consultation, and treatment of urinary tract infection (UTI) and vaginal infection (VI) in women with a history of UTI (*n* = 162) and women with a history of UTI and VI (*n* = 80).

	UTI	VI
	*n* * (%)	*n* * (%)
	***n*** ** = 135**	***n*** ** = 78**
UTI/VI in the past 2 years	102 (75.5)	44 (56.4)
Recurrent UTI **	66 (48.8)	
**Treated for a UTI/VI in the past 2 years**	***n*** ** = 134**	***n*** ** = 78**
No treatment	47 (35.1)	35 (44.9)
1 time	29 (21.6)	18 (23.1)
2 times	23 (17.2)	11 (14.1)
3 or more times	33 (24.6)	13 (16.7)
Unknown	2 (1.5)	1 (1.3)
**Consultation for UWI/VI (multiple answers allowed):**	***n*** **= 162**	***n*** **= 80**
No	57 (35.2)	38 (47.5)
(Classical) Homeopath	29 (17.9)	12 (15.0)
Acupuncturist	14 (8.6)	7 (8.8)
Herbal physician (Western/Eastern)	11 (6.8)	6 (7.5)
Regular GP (with anthroposophic vision ***)	6 (3.7)	3 (3.8)
Regular GP (with homeopathic vision)	5 (3.1)	5 (6.3)
Anthroposophical therapist	5 (3.1)	1 (1.3)
Traditional Chinese physician	4 (2.5)	2 (2.5)
	***n*** **= 131**	***n*** **= 70**
Self-treatment of UTI/VI before or after consulting a health professional	107 (81.7)	48 (68.6)
**Advice on CM/Selfcare for UTI/ VI via (multiple answers allowed):**	***n*** **= 162**	***n*** **= 80**
Internet	68 (42.0)	42 (52.5)
GP/treating physician	34 (21.0)	26 (32.5)
Friends/family	30 (18.5)	10 (12.5)
Social media (blogs, vlogs, Instagram, Facebook)	25 (15.4)	10 (12.5)
Patient associations/fora	10 (6.2)	4 (5.0)
Other:	50 (30.9)	20 (25.0)
-Homeopath	19 (11.7)	4 (5)
-Health shops	7 (4.3)	
-Orthomolecular medicine	7 (4.3)	3 (3.8)
-Own knowledge (through experience)		3 (3.8)

* *n*: women who completed specific section of questions; ** recurrent UTI defined as a minimum of 2 UTIs in the previous 6 months or a minimum of 3 UTIs in the previous 12 months. *** Anthroposophic medicine is an extension of conventional medicine and incorporates a holistic approach to people and nature and to illness [33].

**Table 3:** Self-care and CM for UTI /VI in women with a history of UTI (*n* = 162) and women with a history of UTI and VI (*n* = 80).

	UTI				VI			
	*n*	No Effect	Neutral	Effective	*n*	No Effect	Neutral	Effective
Cranberry juice/cranberry’s	84	20 (23.8)	44 (52.4)	20 (23.8)	^			
Vitamin C	71	21 (29.6)	32 (45.1)	18 (25.4)	^			
D-mannose	53	13 (24.5)	17 (32.1)	23 (43.4)	^			
Probiotics	49	12 (24.5)	27 (55.1)	10 (20.4)	29	9 (31.0)	15 (51.7)	5 (17.2)
Homeopathic remedies	41	5 (12.2)	15 (36.6)	21 (51.2)	11		5 (45.5)	6 (54.5)
Paracetamol/ibuprofen/other painkillers	30	8 (26.7)	15 (50.0)	7 (23.3)				
Acupuncture	11	2 (18.2)	6 (54.5)	3 (27.3)	4		1 (25.0)	3 (75.0)
Chinese/Eastern herbal medicine	5	1 (20.0)	2 (40.0)	2 (40.0)	5	1 (20.0)	2 (40.0)	2 (40.0)
Western herbal medicine	5	1 (20.0)	3 (60.0)	1 (20.0)	1			1 (100)
Anthroposophical remedies *	2		2 (100)		1			1 (100)
Antimycotic cream	^				31	6 (19.4)	11 (35.5)	14 (45.2)
Vaginal shower	^				15	4 (26.7)	11 (73.3)	
Yogurt	^				7	2 (28.6)	3 (42.9)	2 (28.6)
Tea tree oil	^				6	2 (33.3)		4 (66.7)
Honey/honey-containing ointment	^				2		2 (100)	

^: not applicable. * Anthroposophic Medicine is an extension of conventional medicine and incorporates a holistic approach to people and nature and to illness [34]. Due to rounding, percentages do not always add up to 100%.

**Table 4:** UTI/VI interference with daily activities in women with a history of UTI (*n* = 162) and women with a history of UTI and VI (*n* = 80).

		UTI				VI		
		Never	Sometimes	Often		Never	Sometimes	Often
Impact on activities (multiple answers allowed):	*n* *	*n* (%)			*n*	*n* (%)		
Public/social activities	101	14 (13.9)	63 (62.4)	24 (23.8)	60	20 (33.3)	32 (53.3)	8 (13.3)
Going outside	100	18 (18.0)	53 (53.0)	30 (29.0)	58	32 (55.2)	19 (32.8)	7 (12.1)
Sleep	99	17 (17.2)	63 (63.6)	19 (19.2)	60	19 (31.7)	30 (50.0)	11 (18.3)
To be in the mood for activities	97	19 (19.6)	52 (53.6)	26 (26.3)	59	19 (32.2)	31 (52.5)	9 (15.3)
Sports	94	20 (21.3)	51 (54.3)	23 (24.5)	59	21 (35.6)	31 (52.5)	7 (11.9)
Sexual activities	93	18 (19.4)	36 (38.7)	39 (41.9)	58	6 (10.3)	32 (55.2)	20 (34.5)
Work	92	21 (23.1)	49 (53.3)	22 (23.9)	59	24 (40.7)	26 (44.1)	9 (15.3)
Cycling	^				57	14 (24.6)	29 (50.9)	14 (24.6)
**Impact on feelings (multiple answers allowed):**	***n***	***n*** **(%)**			***n***	***n*** **(%)**		
sad/negative	102	31 (30.4)	55 (53.9)	16 (15.7)	59	27 (45.8)	22 (37.3)	10 (16.9)
less confident	101	37 (36.6)	47 (46.5)	17 (16.8)	58	21 (36.2)	24 (41.4)	13 (22.4)
“Why me”	100	49 (49.0)	39 (39.0)	12 (12.0)	58	31 (53.4)	19 (32.8)	8 (13.8)
Annoyance	101	16 (15.8)	51 (50.5)	34 (33.7)	60	8 (13.3)	33 (55.0)	19 (31.7)
Dirty/smell like urine	97	49 (50.5)	38 (39.2)	10 (10.3)	58	17 (29.3)	27 (46.6)	14 (24.1)

^: not applicable. * Number of women who completed the specific answer category. Due to rounding, percentages do not always add up to 100%.

## Data Availability

The data that support the findings of this study are available on request from the corresponding author [EvdW]. The data are not publicly available due them containing information that could compromise research participant privacy/consent.
